# Comprehensive comparison of salivary gland and cutaneous adnexal tumors: analogous versus discrepant clinical, terminological, histological, and molecular features: Part I.: Basic anatomy, overlapping topography, first series of related tumor entities

**DOI:** 10.1007/s00428-026-04454-w

**Published:** 2026-04-14

**Authors:** Stephan Ihrler, Lukas Greber, Abbas Agaimy, Christian Haas, Philipp Jurmeister, Almut Böer-Auer

**Affiliations:** 1 Institute of Pathology, Ludwig-Maximilians-University, Munich, Germany; 2 DERMPATH, Bayerstrasse 36, D-80335 Munich, Germany; 3 Department of Oral and Plastic Maxillofacial Surgery, Military Hospital, Ulm, Germany; 4 Institute of Pathology, Friedrich-Alexander University Erlangen-Nürnberg, University Hospital, Erlangen, Germany; 5 Dermatologikum Hamburg, Hamburg, Germany; 6 Department of Dermatology, Münster University, Münster, Germany

**Keywords:** Salivary gland, Cutaneous adnexal, Tumor, Histogenesis, Molecular pathology, Terminology, Anatomy

## Abstract

The manyfold salivary gland and cutaneous adnexal tumors exhibit a confusing mixture of striking similarities and analogies, but also major discrepancies. Scientific literature focusing on a comparison of these two tumor groups is largely lacking so far. This 2-part review article presents a comparison of both tumor groups, investigating similarities and discrepancies in their clinical aspects, terminology, (immune)-histology, and molecular pathology. An initial comparison of the basic microscopic and functional anatomy of normal salivary and cutaneous adnexal structures is followed by a topographic assessment of practical differential diagnostic difficulties, anatomically highlighted in the overlapping periparotid and perioral areas. Altogether, 21 of 36 benign and malignant salivary tumor entities display histological similarities to adnexal tumors of eccrine, apocrine, and rarely sebaceous, but not trichofollicular differentiation. In this first part of the review on related tumor entities, major histological and partly terminological and/or molecular similarities are compared for salivary pleomorphic adenoma/myoepithelioma versus cutaneous mixed tumor/myoepithelioma, for basal cell adenoma and carcinoma versus spiradenoma, cylindroma, and spiradenocarcinoma, as well as for the principle of secondary malignant transformation ex different types of benign adenomas.

## Introduction

Salivary and skin adnexal glands represent localized or disseminated glandular organs, located at body surfaces, associated to the oropharyngeal mucosa and skin, respectively. Comparing the manyfold salivary gland (*n* = 36) and cutaneous adnexal tumor entities (*n* = 47; according to both WHO classifications [1, 2]), there are, despite major discrepancies, also striking similarities in 21 of the 36 salivary tumor entities (58%) to eccrine, apocrine, rarely sebaceous, but not trichofollicular adnexal tumors. These similarities may relate to clinical aspects, terminology, (immune-)histology, and/or molecular alterations.

The different aspects within related tumor pairs are not necessarily combined, but rather occur independently, seemingly chaotic. Only a few tumor entities show completely identical features in salivary and cutaneous derivation. In the majority, there are histologically similar tumors with discrepant terminology and/or molecular alterations, and, more rarely, tumors with related terminology and/or molecular alterations, but with discrepant histology. The partly bewildering confusion of terminology is mainly consequent to the historically separate development of dermatohistology and general surgical pathology, including head and neck pathology.

The analysis presented herein is partly based on a less thorough review by our group published in the German language in 2014 [3]. In a detailed literature search, we identified comprehensive data separate for salivary and cutaneous adnexal tumors, but no other article, purely devoted to a comparison of both tumor groups was retrieved.

As an unequivocal prerequisite for an optimal understanding, the first part starts with a comparison of embryology and of basic microscopic anatomy of both organ systems. The assignment of tumors to the two different organs is mostly straightforward, according to the defining topography. However, diagnostic difficulties can occur mainly in the periauricular and perioral locations, where both organs anatomically overlap, representing a second major focus in part I.

## Materials and methods

Special knowledge of both tumor groups, and instructive cases for photodocumentation were obtained from experts for salivary gland (S.I., A.A.) and cutaneous adnexal (A.B.) tumors. A systematic literature search was performed in databases of Pubmed and Google Scholar, by use of the program ENDNOTE *(Version X.9.3.3*)*.* A combination of the following keywords was applied: salivary gland, cutaneous adnexal, tumors, terminology, (immune-)histology, molecular pathology, pleomorphic adenoma, and mixed tumor.

### Embryology and microscopic anatomy of salivary and cutaneous adnexal structures

Salivary glands are of ectodermal origin and appear first as epithelial buds in the oral cavity between weeks 7 and 9 of embryonic development. Preacinar buds invaginate the mesenchyme to form cords that develop lumina, followed by extensive branching with formation of terminal secretory units. Cutaneous adnexal structures also develop from the ectoderm at 9–12 weeks with crescent-shaped germinative aggregations that mature to the follicular-apocrine-sebaceous unit and nub-shaped ones that form the eccrine unit. Due to their close embryological relationship, follicular, apocrine, and sebaceous differentiation are often found together in adnexal neoplasms [4].

**Fig. 1:**
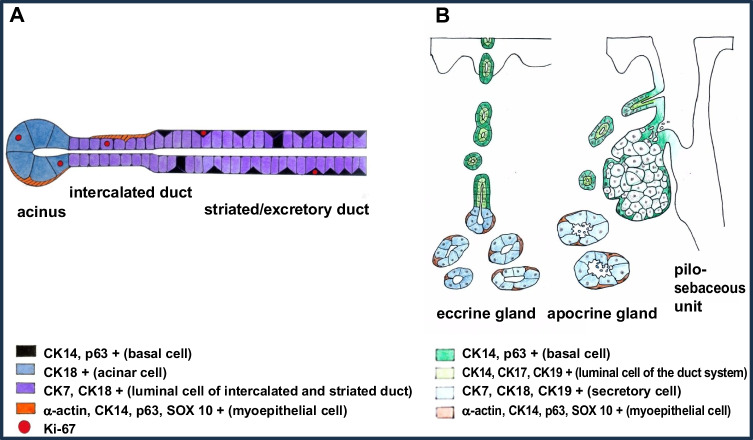
Schematic illustration of microscopic anatomy including immunohistological expression profile of salivary glands (**A** left) and cutaneous adnexal structures (**B** right). **A **The three segments of the salivary duct (striated and excretory ducts combined) are built by 5 different cell types, as shown in the legend (modified according to [5]). **B** The peripheral secretory units of eccrine and apocrine glands (bright blue) are morphologically equivalent to salivary acini (including peripheral contractile myoepithelial cells; orange). The immunohistological expression of the luminal cells of proximal adnexal duct segments (**B**; bright green) expresses basaloid markers, implying suprabasal differentiation, and is thereby discrepant to the secretory differentiation of luminal cells of striated/excretory ducts (**A** purple, CK7/18). The infundibular-follicular differentiation in adnexal structures (pilo-sebaceous unit) is absent in salivary glands, while sebaceous differentiation is rare in salivary glands (not shown)

Features of cutaneous adnexal and salivary gland differentiation are summarized in Fig. 1 and Table 1. A major difference is the restriction of a follicular component to the skin. Therefore, follicular adnexal tumors have no analogues among salivary gland tumors and, hence, a lesion showing features of follicular differentiation cannot be a salivary tumor.

**Table 1:** Comparison of salivary gland versus cutaneous adnexal differentiation

Salivary gland differentiation		Cutaneous adnexal differentiation	
Acini	Acinar cells of either serous, mucous or combined type, abluminal myoepithelial cells	Apocrine glandular	Tubules with decapitation secretion, surrounding myoepithelial layer; variable plasmacytoid, mucinous, squamoid differentiation
		Eccrine glandular	Tubules with mixture of pale and dark cells, surrounding myoepithelial layer
Intercalated ducts	Cuboidal secretory cells devoid of secretory granules, abluminal myoepithelial cells	No analogy	
Striated ducts	Bilayered epithelium with regenerative basal cells and luminal oxyphilic cells	Ducts (apocrine and eccrine)	Two layers of cuboidal cells, luminal cuticula
Excretory ducts	Variable basaloid multilayering		
Sebaceous	Mature vacuolated sebocytes, small sebaceous lobules	Sebaceous	Mature vacuolated and immature sebocytes, sebaceous lobules
No analogy		Follicular	Follicular germinative with or without “germ-and-papilla”-structures; mature follicular: matrical, isthmic, infundibular

A further comparison exhibits important analogies: The salivary acini and intercalated ducts and the end pieces of eccrine and apocrine glands are very similar in cytological, immunohistological, and some functional respects, including the presence of contractile abluminal myoepithelial cells, surrounding the secretory portion (Fig. 1; [3]).

However, the secretory parts also show distinctive differences: In eccrine glands a mixture of clear and dark cells is found. Clear cells produce the watery composition of sweat, while dark cells produce glycoproteins. The secretory (acinar) part of apocrine glands has larger, columnar, eosinophilic cells, which produce oily secretion, being transferred into the lumen by apocrine/decapitation secretion [4]. In comparison, salivary acini are either purely serous (watery secretion; parotid gland), purely mucous (viscous secretion; sublingual and minor glands), or combined (submandibular gland; Table 1 [3]).

The principal composition of salivary striated/excretory and eccrine/apocrine ducts is identical with the presence of two layers of cuboidal cells: darker basal abluminal cells and paler suprabasal luminal cells (Fig. 1) [3, 6, 7]. However, there is a fundamental difference in the luminal cell component: The luminal oxyphilic cells of salivary ducts are of glandular type and hence secretory active, which correlates with the expression of secretory-type cytokeratins 18/7 [8]. In contrast, luminal cells of eccrine ducts are involved in electrolyte reabsorption, but not secretion, while no such function is known for apocrine ducts. Interestingly, luminal cells in eccrine and apocrine ducts express CK14 like basal cells (CK18/7 negative), which could be interpreted as a more stable basal cell phenotype (Fig. 1) [3, 9]. These discrepancies have not yet been highlighted in the literature. Our group assumes that they may relate to differences in the anatomical location (deep, protected soft tissue in salivary glands versus superficial dermal location in cutaneous adnexae, exposed to adverse external influences).

A further distinction exists with regard to sebaceous glands. In the skin, sebaceous glands are found at every hair follicle [3]. The lobulated architecture develops under the influence of estrogen/androgen, while in the absence of such influence, they are thin strands. They connect to infundibula with a short duct (Fig. 1). In salivary glands, inconspicuous sebaceous glands are located at the transition of intercalated to striated ducts; however, being so rare that they are not evident in routine histology [3, 10].

The distinction of cutaneous adnexal tumors into neoplasms with eccrine versus apocrine differentiation has no counterpart in salivary glands. [1, 11]. But even in the skin, differentiation between tumors of putative eccrine and apocrine origin may be difficult and sometimes impossible, if decapitation secretion or associated follicular or sebaceous differentiation are absent.

It is still unclear in both organ systems whether the complex tumorigenesis derives from certain differentiated anatomical cell types or ductal segments, or generally from hypothetical stem cells. With respect to salivary glands, previous hypotheses on physiological proliferation/regeneration [5, 8] have been strongly substantiated recently by experimental data, which show that differentiated cell types (like acinar cells) regenerate by themselves without contribution by stem cells [5, 10]. Stem cells have been postulated in eccrine and apocrine glands, but it is not known, whether they are involved in tumorigenesis.

### Topographical hotspots of differential diagnosis between salivary and cutaneous adnexal tumors

**Fig. 2:**
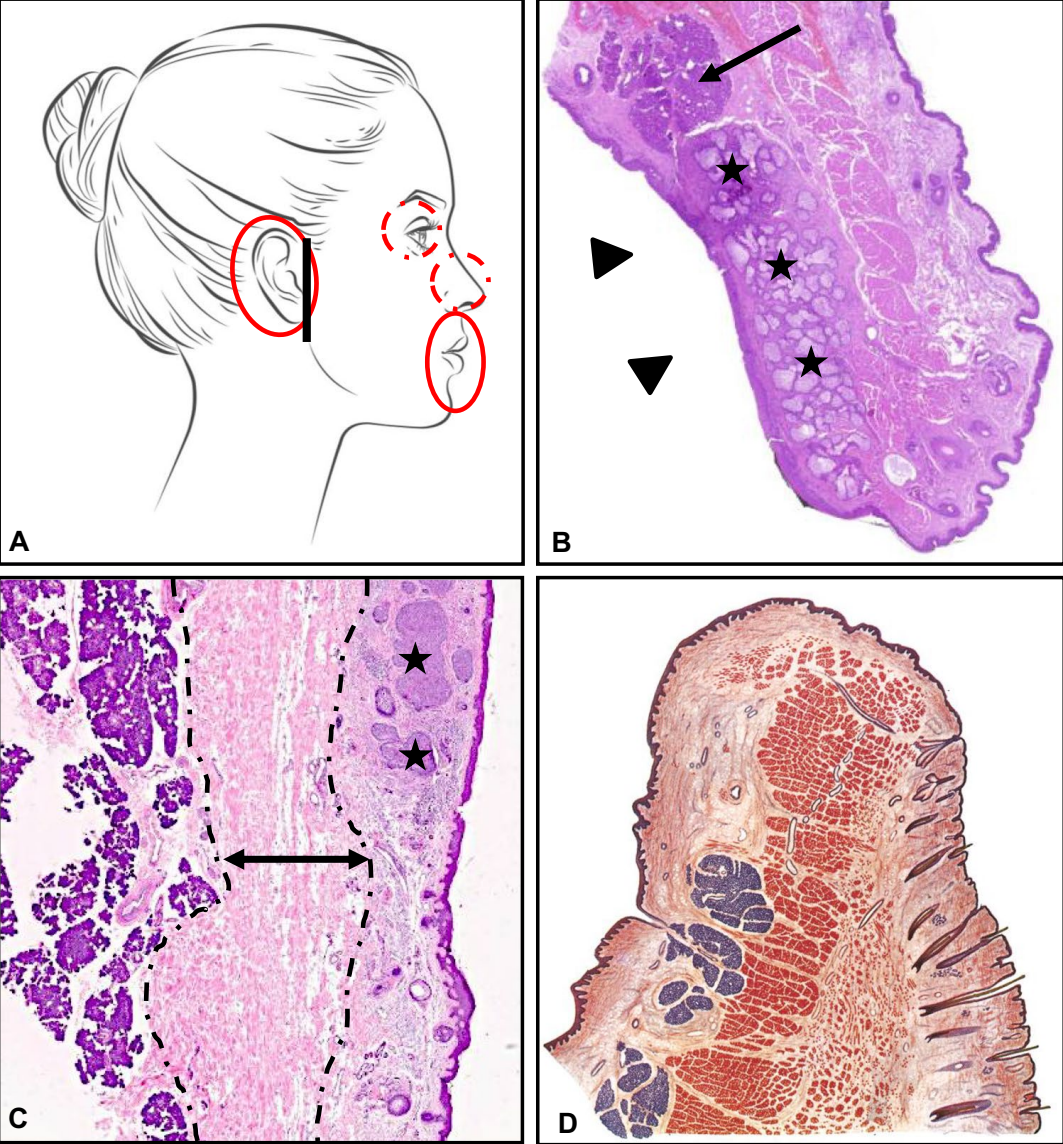
**A** Areas of potential topographical overlap of salivary and adnexal tumors. **D** The perioral area corresponds to the upper and lower lip. Section through lower lip with skin and adnexal structures (right) and mucosa with minor lip glands (violet; left), in between the musculus orbicularis oris (red) [12]. **C** Histological section from preauricular (corresponding to black line in **A**) with skin and basal cell carcinoma (stars;right) and superficial part of the parotid gland (left). In between a “soft tissue bridge” (arrow), devoid of epithelial structures, measuring 3–4 mm. The periauricular area may be terminologically confusing, as it may be clinically called “pre-auricular” (or “cheek”), “infra-auricular” (or “retro-mandibular”), or “retro-auricular” (in reality: behind the earlobe). **B** Histological section through the upper eyelid. On the conjunctional side (left; arrowheads) admixture of sebaceous glands (Meibom; stars) with rare salivary glands (“Gll. tarsales”; arrow). Further rare areas of overlap are in the vestibulum nasi and in the ear canal (**A**)

The necessity of a differential diagnostic distinction between histologically similar salivary and cutaneous tumors is restricted mainly to the periauricular and perioral, rarely periocular location, where both groups topographically overlap (Fig. 2) [3].

#### Topographical overlap in the periauricular area

In surgical resections of the parotid area, we measured the average distance between the deepest cutaneous adnexal structures and superficial components of the parotid gland. The resulting subcutaneous soft tissue area, devoid of adnexal and salivary structures, measures in paraffin-embedded slides only 3–4 mm (Fig. 2C).

Cutaneous adnexal tumors are frequent in the head/neck area, including the periauricular region [1] and they may grow deep into the subcutis and rarely into the parotid gland*.* On the other hand, the majority of tumors of the parotid gland develop in the lateral lobe; malignant tumors may extend into the periparotid subcutaneous fat and may even infiltrate the dermis. In addition, as the parotid gland is not encapsulated, during growth, both benign and malignant tumors may be partially or completely “pushed out” of the relatively firm gland into the particularly soft subcutaneous fat. By this mechanism, a parotid tumor may completely be located outside of the gland and may clinically and histologically be misinterpreted as a deeply located cutaneous adnexal tumor.

In such instances, pure topographical location alone does not allow assignment of a tumor to the skin adnexa or parotid gland. This kind of challenging differential diagnosis typically occurs in the preauricular and infraauricular (= retromandibular) area (Fig. 2A, C). In the latter location, lesions are clinically often misdiagnosed as lymph nodes, lipomas, or cysts [13] and they are hence frequently treated inadequately, not by experts of parotid surgery, which may lead to adverse recurrences (see chapter pleomorphic adenoma).

**Fig. 3:**
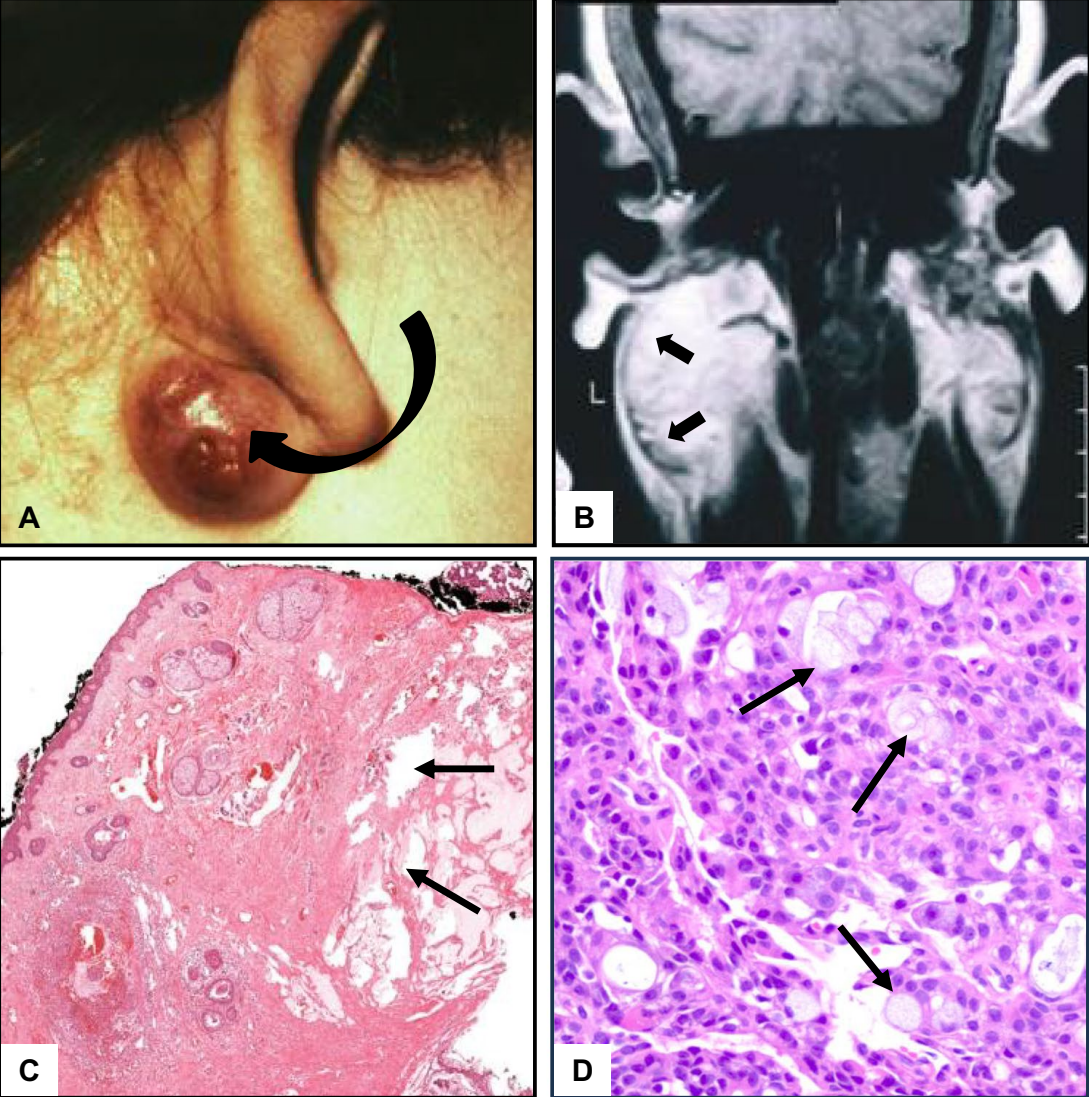
**A** Nodule posterior to the ear lobe (clinical description: “retroauricular”): Not infrequent clinical manifestation of parotid tumors, here mucoepidermoid carcinoma. **B** Corresponding magnetic resonance image of this parotid tumor (arrows; permission due to [3, 13]). **C** Own case of a parotid mucoepidermoid carcinoma approaching dermal structures (arrows). **D** Histology of mucoepidermoid carcinoma, mucoid cells marked by arrows

In the experience of the authors, parotid tumors not infrequently clinically present as swellings posterior to the ear lobe, obviously because the subcutaneous fat is soft and provides little resistance to a growing tumor [13]. In the absence of a clinical photo, the imprecise clinical designation “retroauricular” may tempt an incorrect histological diagnosis of a skin adnexal tumor, because “salivary tumors do not occur in the retroauricular area”. The illustrated case of a parotid mucoepidermoid carcinoma, clinically indicated as “retroauricular” (in reality: posterior to the ear lobe), highlights this situation (Fig. 3A). In the absence of adequate clinical information, a final diagnosis should be postponed in case of a difficult tumor, as this constellation bears the risk of misdiagnosis with severe consequences [3, 13].

#### Topographical overlap in the perioral area (lips)

**Fig. 4:**
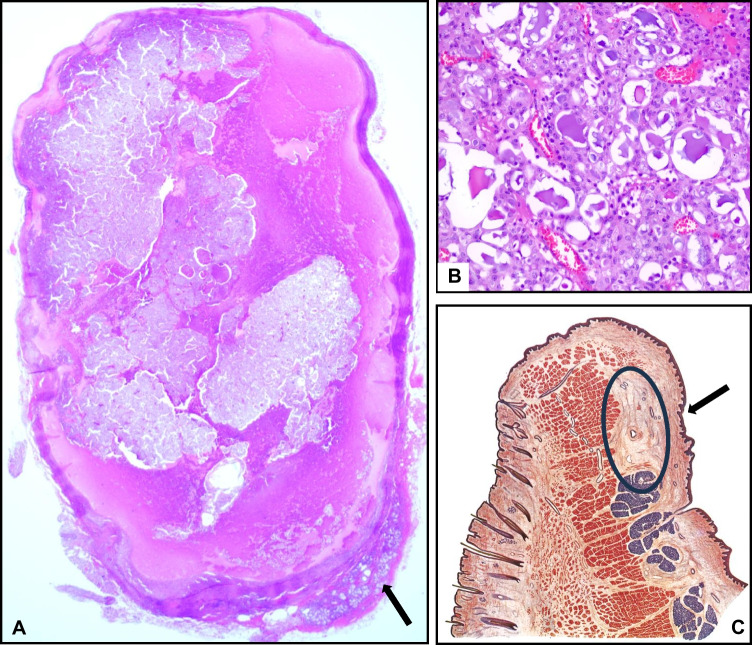
**A** Enucleation from the lower lip of a well circumscribed, solid-cystic tumor with adjacent minor salivary gland (arrow). **B** In high-power micro-cystic glandular proliferation with eosinophilic intracystic secretion, no cellular atypia. Diagnosis: secretory carcinoma (with translocation *ETV6::NTRK3*). **C** Putative topographical position of the tumor. Attribution of this secretory carcinoma to a lip salivary gland due to adjacent minor gland (**A**, arrow) and due to resection from the mucosal site by ENT-surgeon (arrow)

The lips represent an anatomically unique constellation, as cutaneous adnexal structures and minor labial salivary glands are tightly neighbouring, only separated by inconstant bundles of the musculus orbicularis oris (Fig. 2D). Lip tumors originating from labial glands tend to be more adjacent to the mucosa and are therefore typically operated on from the mucosal site (with or without a mucosal component) by ear-nose-throat or maxillo-facial surgeons. This constellation is illustrated by the case of a secretory carcinoma of lip salivary glands (Fig. 4).

**Fig. 5:**
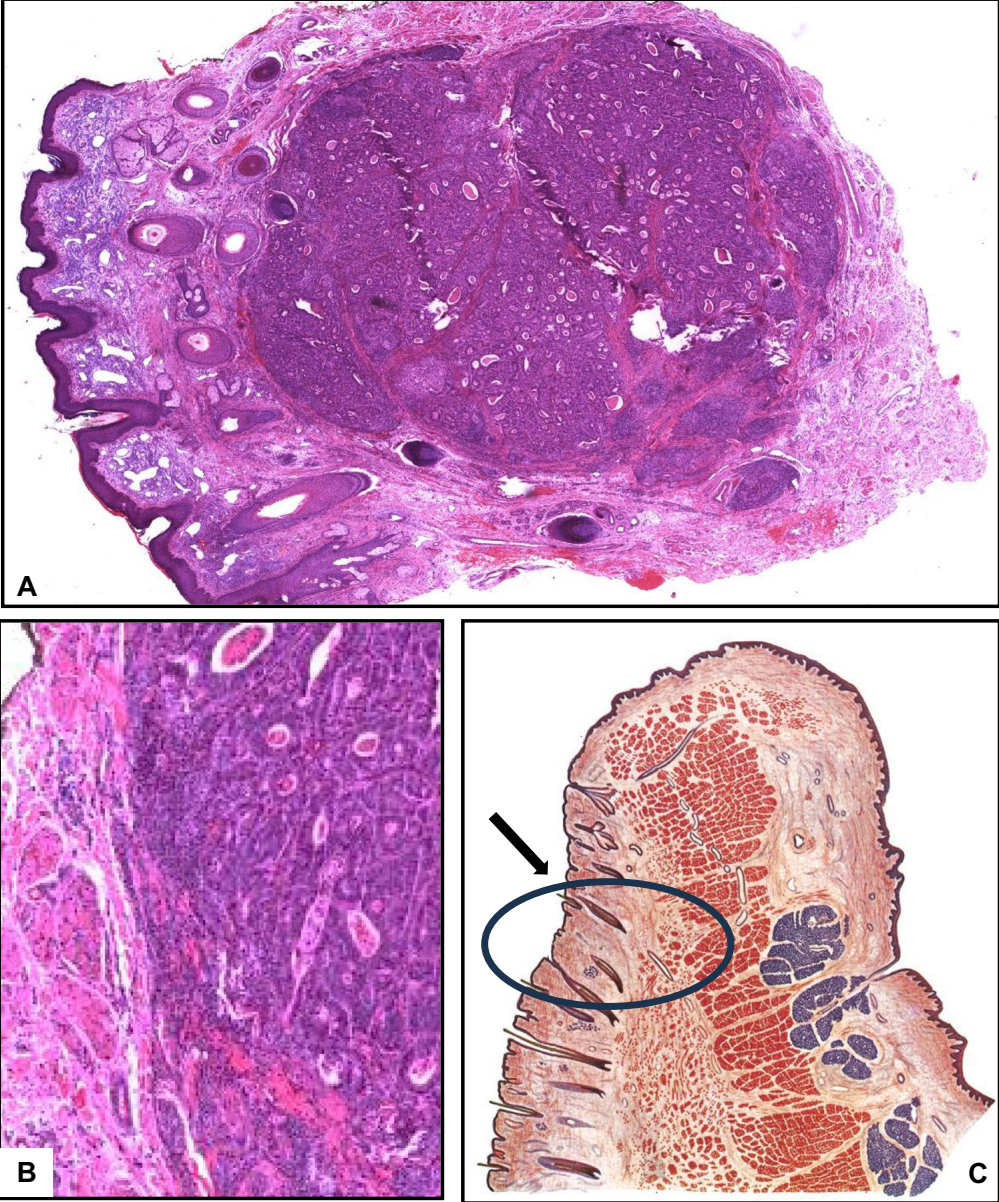
**A** Resection from lower lip with relatively well circumscribed, solid-glandular tumor including skin excision. **B** Higher magnification of a glandular tumor without cellular atypia. **C** Topographical position of the resection, performed from the skin site by dermatologist (arrow). Diagnosis: Endocrine mucin-producing sweat gland carcinoma

Lip tumors originating from cutaneous adnexa, however, tend to be more adjacent to the skin and are therefore typically operated from the skin site, frequently with a skin excision, and mostly by dermatologists. This constellation is illustrated by the case of an endocrine mucin-producing sweat gland carcinoma (Fig. 5).

**Fig. 6:**
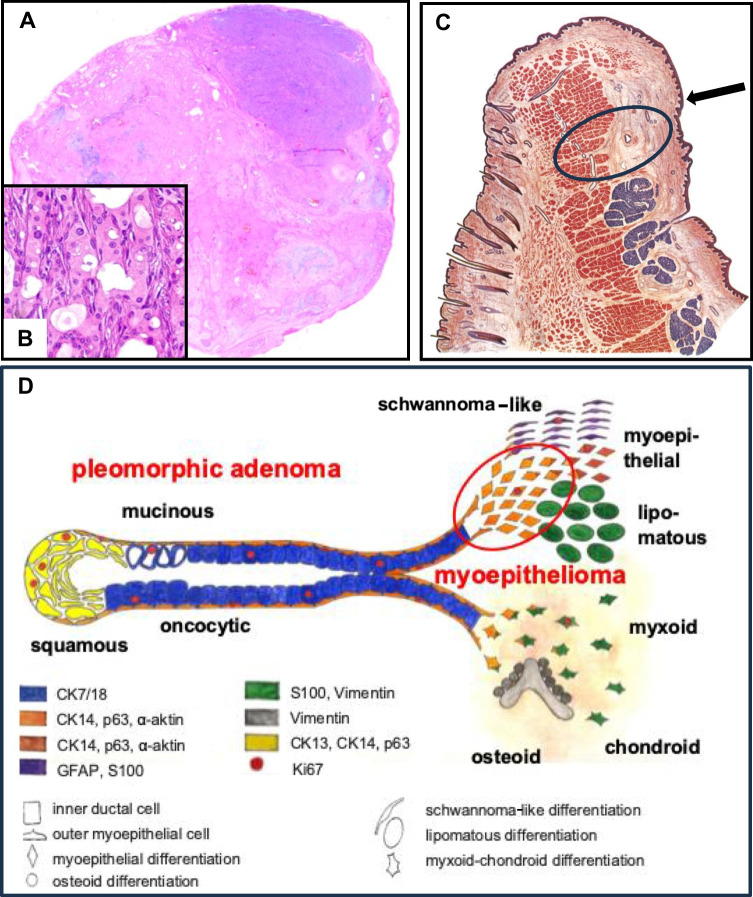
**A**, **B** Well-circumscribed tumor with “pleomorphic” epithelial and stromal differentiation including myxoid-chondroid features, histologically either PA or apocrine mixed tumor. Devoid of skin, mucosa, and salivary/adnexal structures. **C** Putative topographical position of the tumor, due to resection from mucosal site by ENT-surgeon (arrow). Diagnosis: PA of lip minor gland, due to clinical information and absence of follicular differentiation. **D** Schematic illustration of the complex differentiation pattern of salivary PA (modified according to [14]). The pathogenetic starting point is biphasic tubules (centre). Parallel to loss of luminal ductal cells (blue), the abluminal myoepithelial cells (orange) undergo a progressive “epithelial-mesenchymal transdifferentiation” into variably combined schwannoma-like, myoepithelial (if exclusive: entity of myoepithelioma), lipomatous, myxoid, chondroid, and rarely osteoid phenotypes (right). More rarely, diverse epithelial metaplasias (mucinous, oncocytic, squamous) may occur (left)

Enucleated lip tumors without any component of skin, mucosa, adnexal structures, or minor salivary glands may be difficult to assign to the two organ systems, if the histological presentation is not entity or organ specific. In this situation, the site of operation (skin versus mucosa) may be a helpful clinical clue. This is illustrated by the case of a pleomorphic adenoma, operated from the mucosal site (histologically identical to apocrine mixed tumor; Fig. 6A–C).

The histological diagnosis of minor salivary gland tumors is more challenging according to a recently published investigation of our group [15]. As one of the most important reasons, the fact was identified that carcinomas of minor glands (in contrast to major glands) typically do not exhibit classical signs of malignancy, as they are almost regularly highly differentiated, do not show increased proliferation, and often no infiltration, as demonstrated in the case of a secretory carcinoma of the lip (Fig. 4). An analogous situation may complicate the diagnosis of cutaneous adnexal tumors, as demonstrated in the case of an endocrine mucin-producing sweat gland carcinoma in the lip, which also lacked histological criteria of malignancy (Fig. 5). Such a difficult differential diagnosis is not limited to the question “tumor of salivary or cutaneous origin,” but may be severely complicated by the frequently difficult question “benign or low-grade malignant.”

#### Rare topographical overlaps in further areas

Cutaneous adnexal tumors of the eyelids may topographically overlap with salivary tumors in the conjunctiva (mostly oncocytoma). Malignant tumors of the deep-seated lacrimal gland rarely infiltrate so extensively that they manifest in conjunctiva or adjacent skin. The vestibulum nasi may rarely show an overlap of adnexal and salivary tumors. The external auditory canal is typified by numerous ceruminous glands (modified apocrine glands) and therefore a dominance of cutaneous adnexal tumors (mostly apocrine mixed tumor) is not surprising. As a rare differential diagnosis, parotid tumors (typically malignant) may infiltrate into the ear canal (Fig. 2).

In the following chapters, the first series of related pairs of tumors of both organ systems are compared.

### Salivary pleomorphic adenoma/myoepithelioma versus cutaneous mixed tumor/myoepithelioma

Salivary pleomorphic adenoma (PA) and the apocrine type of cutaneous mixed tumor represent one of the most interesting couples of corresponding tumor types. They exhibit major similarities with respect to (immuno-)histological and molecular features, while there are significant differences in clinical characteristics and terminology (Table 2) [19]. 

**Table 2:** Correlation between salivary pleomorphic adenoma and cutaneous apocrine mixed tumor

	Pleomorphic adenoma [14, 15]	Apocrine mixed tumor [1, 4, 16–18]
Frequency	Most frequent salivary tumor	Common
Average age	43 years	53 years
Site	Most frequently parotid gland	Skin of head and neck mostly
Average size	15–20 mm	10 mm
Gender ratio (men:women)	1:1	2:1
Structural variation	Vast structural and cellular diversity (“pleomorphic”)	Broad spectrum with follicular, sebaceous, myoepithelial differentiation
Myoepithelioma	Presumably end of a continuum of PA	Presumably end of a continuum of apocrine mixed tumor
Immunohisto-chemistry	Biphasic: abluminal (p63, SOX10) + luminal (CK18)	SOX10, p63, S100, calponin, SMA, cytokeratins, PLAG1, Follicular differentiation: BerEP4, PHDLA1
Molecular alterations	Translocations with *PLAG1* or *HMGA2*	Translocations with *PLAG1* or *HMGA2*
Treatment	Complete (“extracapsular”) resection	Enucleation usually sufficient
Risk of recurrence	Not infrequently multinodular recurrences	Rare recurrences (not multinodular)
Malignant transformation	Rather frequent	Very rare

**Fig. 7:**
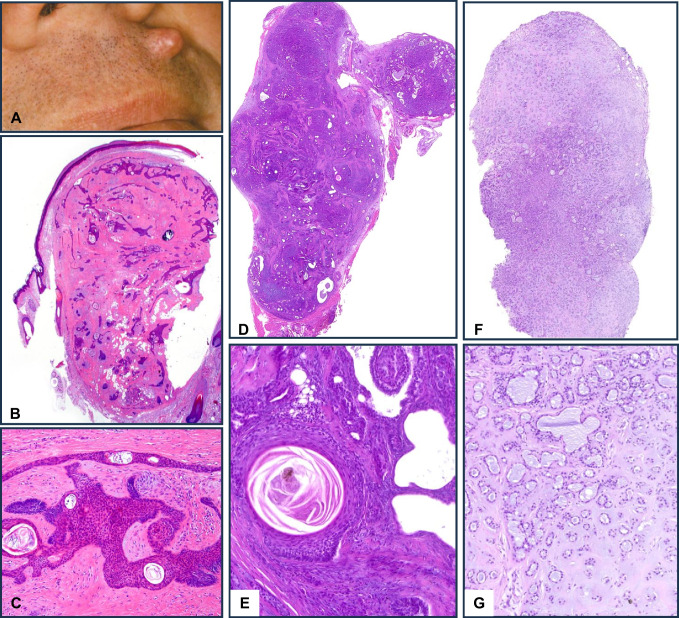
Cutaneous mixed tumor. **A**–**C** Apocrine mixed tumor located at the upper lip and excised from the outside by a dermatologist, vertically oriented and well circumscribed with interconnected, bilayered tubules and small cysts, some with keratinization. **C** Basophilic palisaded cells represent follicular germinative differentiation. **D**, **E** Excision of a well circumscribed lesion from the lower lip, excised from the outside by a dermatologist. No salivary glands are seen and the lesion shows obvious follicular germinative differentiation with cysts and pigmented keratin, together with elongated tubules, indicating apocrine mixed tumor. **F**, **G** Excision of a well circumscribed lesion from the scalp. Chondroid stroma and monomorphic short tubules and microcysts, typically with one cell layer, indicate eccrine mixed tumor

The term “pleomorphic” in PA refers to these multiple structural and cytological differentiations, not to cellular atypia. Unlike salivary PA, cutaneous mixed tumors are still separated into those of apocrine and eccrine differentiation. The apocrine type is by far more common and displays more similarities with PA, since the epithelial component shows typically elongated and branching biphasic tubules and apocrine (“decapitation”) secretion (compared to eccrine mixed tumor, which has short tubules [20]). In contrast to PA, prominent follicular and/or sebaceous elements are often found in apocrine mixed tumors (Fig. 7).

Recent molecular data strongly indicate that apocrine and eccrine types of mixed tumor are separate entities. While translocations involving the *PLAG1*- and *HMGA2*-genes dominate in apocrine mixed tumors, eccrine mixed tumors frequently show internal tandem duplications of SOX10 [16]. In addition, new methylation data demonstrate separate clusters for apocrine and eccrine tumor types, suggesting separate entities; however, a concept not yet accepted [16].

There is a second confusing constellation: PA displays a vast histological continuum from cell-rich to cell-poor, myxoid-chondroid variants. This continuum includes myoepithelial-rich variants, rather arbitrarily separated as salivary myoepithelioma (Fig. 6D) [14]. An analogous continuum has been proposed in the skin, ranging from apocrine mixed tumor with a variable amount of myoepithelial differentiation to pure myoepithelioma [19]. In the skin, by definition, the diagnosis of myoepithelioma currently requires the absence of tubular elements, while a proportion of up to 5% tubular structures still permits the diagnosis of salivary myoepithelioma [21].

Molecular data demonstrate frequent *PLAG1* and, more rarely, *HMGA2* gene rearrangements both in apocrine mixed tumors [22] and in salivary PA, as well as in cutaneous and salivary myoepitheliomas. In a recent methylation study of our group, this concept is strongly supported for salivary tumors, demonstrating a common cluster for PA and myoepithelioma [23]. Together with overlapping (immuno-)histological features, these molecular data imply a wide, continuous morphological spectrum for both, cutaneous apocrine mixed tumor/myoepithelioma and for salivary PA/myoepithelioma [16, 17]. Therefore, changes in tumor classification and terminology are expected in this area in the future.

**Fig. 8:**
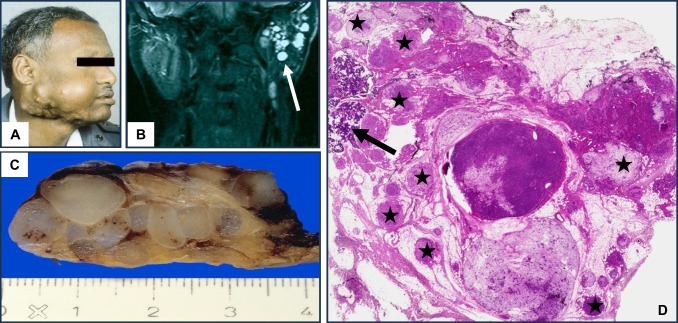
Multinodular recurrence of PA. **A** Clinical situation and **B** magnetic resonance imaging with multiple and widespread subcutaneous and deeper nodules (arrow). **C** Operative specimen with multiple small to medium-sized nodules. **D **Histology with numerous very small (stars) to medium-sized, cell-poor to cell-rich nodules of recurrent PA. Salivary parenchyma on the left (arrow)

An astonishing, so far totally unclear, but clinically most relevant discrepancy is the observation that cutaneous mixed tumors are practically harmless [24], while PAs may produce severe clinical complications [14]. Pure enucleation of PA (frequently performed by unexperienced surgeons) may cause repeated multinodular recurrences (Fig. 8) with increased risk of the inherent danger of malignant transformation (see chapter malignant transformation). While myoepithelial carcinoma, the malignant counterpart of myoepithelioma, is very rare in the skin (usually de novo development) [1], salivary myoepithelial carcinoma is much more frequent and is in half of the cases developing secondarily ex PA, with an unpredictable, often dismal prognosis [25].

### Salivary basal cell adenoma versus cutaneous spiradenoma/cylindroma

**Fig. 9:**
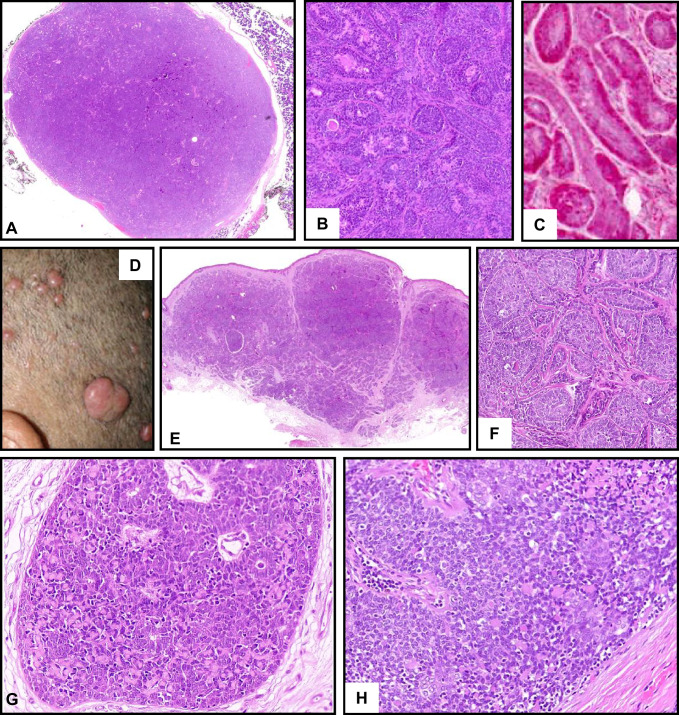
Histological similarity of salivary basal cell adenoma (**A**, **B**, **C**, **G**) and cutaneous cylindroma/spiradenoma (**D**, **E**, **F**, **H**). **A** Basal cell adenoma, well circumscribed nodule in the parotid gland. **B** Jigsaw-puzzle pattern of nodules and tubules with thickened basement membrane (membranous type). **C** Intense cytoplasmic and nuclear staining for beta-Catenin. **D** Patient with Brooke-Spiegler Syndrome. **E**, **F** Cylindroma with pseudoinfiltrative, jigsaw-puzzle pattern and thickened basement membrane. **G** Salivary basal cell adenoma with solid pattern, multiple hyaline globules, and infiltrating lymphocytes. **H** Practically identical pattern in cutaneous spiradenoma with intense infiltrating lymphocytes

Salivary basal cell adenoma and cutaneous spiradenoma/cylindroma are benign neoplasms with similar (immuno-)histological presentation, but with different terminology and molecular alterations. Both consist of two types of cells, monomorphic basaloid cells similar to basal cells of salivary striated ducts (hence the term basal cell adenoma) or peripheral cells of apocrine ducts, respectively, and ductal luminal cells with a more eosinophilic appearance, typically without or rare lumen formation (Fig. 9).

In basal cell adenoma, four architectural patterns have been separated, namely, solid, trabecular, tubular, and membranous [26], often blending with each other. Morphological features of spiradenoma and cylindroma are overlapping [4]. The most striking resemblance exists between the membranous type of basal cell adenoma and cylindroma, as both have a jigsaw puzzle pattern of closely arranged nodules. The solid pattern in basal cell adenoma is more reminiscent of spiradenoma. The epithelial formations are encircled by trabecular and/or globular eosinophilic, PAS-positive basement membrane material. This may be so extensive that it replaces lobules. While spiradenomas usually have less rimming of basement membrane, they may assume the appearance of “pseudorosettes.” So-called peppering with CD3+ lymphocytes and CD1a+ Langerhans cells is a diagnostic hallmark of spiradenoma, while this is less pronounced and less well recognized in basal cell adenoma (Fig. 9G, H) [27].

Immunohistochemistry highlights the biphasic basaloid-luminal pattern. The paler luminal cells stain for CK18, CK7, CEA, and EMA; the basaloid cells with basal/myoepithelial markers such as actin, calponin, S100, p63, p40, and SOX10. CK18-positive tubules with lumina are found in variable density in basal cell adenoma and in cylindroma/spiradenoma, but they rarely are numerous, except in the tubular variant of basal cell adenoma. Cylindroma/spiradenoma may have signs of apocrine (decapitation) secretion and may show follicular or sebaceous differentiation. Myxochondroid changes are absent (in contrast to PA and mixed tumors). Basal cell adenoma and cylindroma are usually asymptomatic, while spiradenoma may be painful on palpation, which has been associated with thrombotic vessels.

Alteration of the *CYLD* gene is very frequent in cylindroma and is found in about 1/3 of spiradenoma, while a missense mutation in the ALPK1 gene is found in ½ of spiradenoma [28]. Basal cell adenomas show in about 80–95% nuclear overexpression of beta-Catenin (Fig. 9C), not perfectly correlating to mutation of the *CTNNB1*-gene (in 60%), but also mutation of the *CYLD* gene in 36% [26, 29]. Current data indicate that the conventional (non-membranous) type of salivary basal cell adenoma is not only morphologically, but also genetically (mostly *CTNNB1*-altered) distinct from the membranous type (mostly *CYLD1-*altered); only the latter is probably related to adnexal spiradenoma/cylindroma. 

A rare specific cutaneous manifestation of cylindroma is the so-called turban tumor, that develops on the scalp by confluence of many nodules in Brooke-Spiegler Syndrome (Fig. 9D), which is caused by a heterozygous mutation in the *CYLD* gene on chromosome 16q12 (OMIM-P 605041) [30]. Allelic disorders include familial cylindromatosis (OMIM-P 132700) and multiple familial trichoepithelioma-1 (OMIM-P 601606). The membranous type of salivary basal cell adenoma has also been observed in Brooke-Spiegler Syndrome and an identical loss of heterozygosity on chromosome 16q 12–13, the region of the *CYLD* gene, has been described in membranous basal cell adenoma and cylindroma [31].

The pathogenesis of the rare malignant counterparts seems to be totally different: Salivary basal cell adenocarcinoma develops de novo in the great majority of cases and the differential diagnosis between basal cell adenoma and well-differentiated basal cell adenocarcinoma may be difficult and may purely rely on unequivocal infiltration. On the contrary, the majority of spiradenocarcinomas develop secondarily from preexistent spiradenomas [32]. Importantly, the frequent (pseudoinfiltrative) multinodularity in otherwise benign cutaneous adnexal neoplasms, especially in cylindroma and spiradenoma, should not be misinterpreted as infiltrative growth pattern [1].

### Malignant transformation ex various types of primary adenomas

**Fig. 10:**
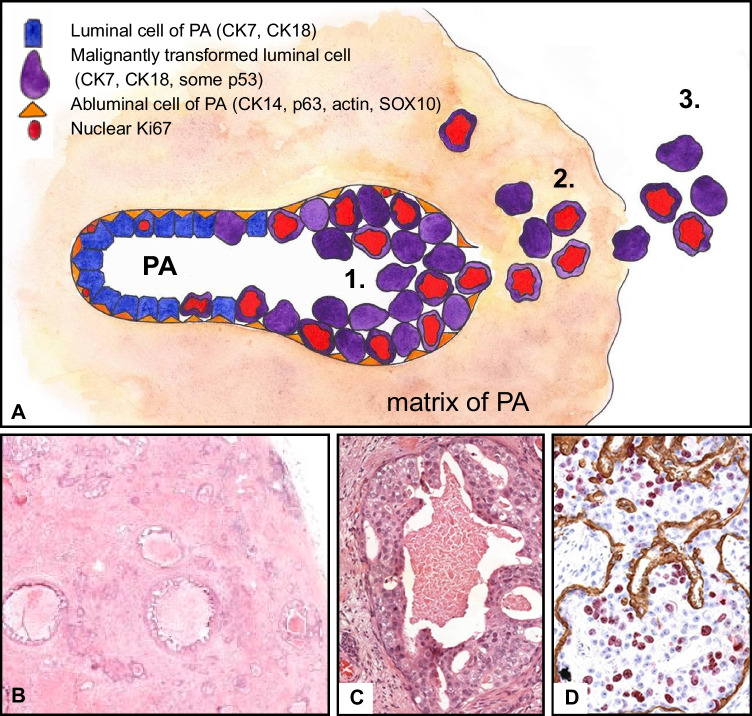
**A** Schematic illustration of the multistep carcinogenesis of carcinoma ex PA [25]. (1) Increased proliferation (Ki67: nuclear red) with intratubular expansion of malignantly transformed luminal cells (excellent prognosis). (2) Rupture of basement membrane and infiltration into the matrix of PA (good prognosis). (3) Infiltration beyond pseudocapsule of PA into surrounding tissue (bad prognosis in case of extensive invasion of >6 mm). **B**, **C** High-grade cribriform intraductal malignant transformation within tubuli of PA with comedo-type necrosis and high-grade proliferation (**D**; Ki67: red), surrounded by preexisting myoepithelial cells of tubuli of PA (CK14: brown; corresponding to stage 1 in A)

Secondary malignant transformation is relatively frequent in PA (carcinoma ex PA), representing the fifth most frequent salivary carcinoma entity. The most frequent variant is salivary duct carcinoma ex PA, typically with positivity for the androgen receptor, followed by myoepithelial carcinoma ex PA. Carcinoma ex PA is characterized by dismal prognosis in advanced stages with extensive extracapsular invasion (about 50% of cases), while early stages are limited to intraductal/intracapsular carcinoma with very good prognosis. Our group has contributed to a better understanding of this prognostically and therapeutically highly relevant multistep carcinogenesis (Fig. 10) [25, 33].

In contrast, secondary malignant transformation altogether occurs less frequently, however, in a wider spectrum of benign adnexal tumors [24]. Spiradenocarcinomas and cylindrocarcinomas both tend to develop from their benign counterparts. Cylindrocarcinomas occur especially in the setting of longstanding lesions in Brooke-Spiegler Syndrome/familial cylindromatosis. High proliferation rate (Ki67), increased and atypical mitoses, infiltrative growth, perineural involvement, and *TP53* mutation indicate malignant transformation [34, 35]. The major difference is that secondary carcinogenesis (carcinoma ex PA) represents true malignant transformation in the salivary glands, while it represents a morphological continuum between benign and malignant in the skin adnexa, with many cases being difficult to separate into benign versus bona fide malignant.

The above explained principle of intraductal or intracapsular carcinoma ex PA as initial step of malignant transformation (Fig. 10) has been documented casuistically in carcinoma ex spiradenoma (and ex hidradenoma), but since the prognosis of spiradenocarcinoma is much more favourable than that of carcinoma ex PA, it is clinically less important [3, 36].

#### Outlook to part II

Part II of the comprehensive comparison of salivary and cutaneous adnexal tumors will cover a further series of histologically similar pairs of tumors, including secretory/microsecretory adenocarcinoma, adenoidcystic carcinoma, mucinous/mucoepidermoid tumors, sebaceous tumors, and lymphoepithelial tumors. Finally, it will present a synoptic comparison of general aspects like intraductal precursor lesions, grading of malignancy, the relevance of immunohistochemistry and of molecular pathology, as well as of aspects of multifocality and of inherent genetic syndromes.
